# The relationship between self-oriented perfectionism and exercise participation: based on the dualistic model of passion

**DOI:** 10.3389/fpsyg.2024.1373844

**Published:** 2024-06-25

**Authors:** Weipeng Zhang, Yuanjiao Zhu, Feng Jiang, Huitao Song

**Affiliations:** ^1^School of Sports Sciences, Tianjin Normal University, Tianjin, China; ^2^Tianjin University of Technology and Education, Tianjin, China; ^3^School of Business Administration, Jeonbuk National University, Jeonju, Republic of Korea

**Keywords:** self-oriented perfectionism, passion, harmonious passion, obsessive passion, exercise participation

## Abstract

**Background:**

During the critical formative years of college, active participation in sports not only helps to alleviate stress, but also promotes the development of healthy habits. Although the multifaceted benefits of exercise have been widely recognized, there is a relative dearth of research on the relationship between personality traits, particularly college students’ self-oriented perfectionism (SOP), and exercise participation.

**Methods:**

A questionnaire survey of 374 college students was conducted using the snowball sampling method. SPSS 26.0 and Mplus 8.3 were employed in this study to analyze the correlations between the variables, and on this basis, the effect of SOP on exercise participation was examined. The study also used 5,000 bootstrap samples and a 95% bias-corrected confidence interval to test the significance of the mediating effects.

**Results:**

Correlation analysis showed that SOP was positively correlated with exercise participation. Harmonious passion and obsessive passion were positively correlated with SOP, and exercise participation. Further, the results of structural equation analysis revealed that SOP increased exercise participation. Harmonious passion and obsessive passion positively mediated the effect between SOP and exercise participation, respectively.

**Conclusion:**

This study provides new perspectives to better understand college students’ exercise participation, emphasizing the importance of SOP and its influence on exercise participation through harmonious and obsessive passions. These findings have important implications for the development of effective exercise promotion strategies.

## 1 Introduction

The university stage is a critical period in personal development (San Román-Mata et al., 2020), when academic pressures, career planning challenges, and relationship changes combine to affect the physical and mental health of college students (Iqbal et al., 2023). In this context, exercise participation is particularly important. Research has shown that regular exercise participation not only helps individuals regulate their body weight and body fat percentage and prevent obesity (Brzuszkiewicz et al., 2022; Gomes et al., 2023), but also has a positive impact on college students’ resilience (Zach et al., 2021), emotional intelligence (San Román-Mata et al., 2020) and mental health (Ma et al., 2021). Despite the benefits of exercise participation to an individual’s physical and mental health, college students still face a number of challenges in adhering to exercise participation, including academic pressures, time constraints, and fewer available fitness classes (Pellerine et al., 2022). Therefore, it is critical to explore the motivations for promoting exercise participation among college students and to integrate physical activity into their daily lives to promote their physical and mental health.

Prior research has shown that exercise participation is mainly influenced by a variety of factors such as individual characteristics, environmental characteristics, and exercise traits (Teunissen et al., 2023). For example, goal setting can increase an individual’s exercise participation (Swann et al., 2021). A safe and well-resourced community environment provides residents with more opportunities to exercise and stimulates their interest in participating in physical activity (Zhang et al., 2022). Although previous scholars have explored the antecedents that promote exercise participation from different perspectives, few scholars have examined the relationship between college students’ tendency toward SOP and exercise participation behaviors.

SOP represents a self-demanding tendency to evaluate and define oneself by establishing high standards and striving for perfection (Verner-Filion and Vallerand, 2016). This pursuit of perfection is not only reflected in a number of domains, such as academic and social activities (Bong et al., 2014; Nepon et al., 2016), but is even more pronounced in physical activity, where it can greatly influence an individual’s exercise behavior and performance. Many sports psychologists have been keenly interested in how athletes’ perfectionism affects exercise performance (Ahmed et al., 2021). Prior research has shown that college students with SOP tendencies seek perfection and have higher persistence and achievement motivation (Vink and Raudsepp, 2018). We believe that this characteristic of SOP will be a driving force for university students to motivate them to set and pursue perfection in their sport and to participate more actively in sports.

To better understand the relationship between SOP and exercise participation, this study also considered the role of passion in their relationship. Passion is a strong inclination toward activities that people enjoy, that they consider important, and that they invest time and energy in, and is a central part of one’s identity (Yukhymenko-Lescroart, 2022). According to the dualistic model of passion, there are two types of passion, harmonious and obsessive passion, which stem from intrinsically true desires and are aligned with an individual’s values and goals, whereas obsessive passion manifests itself as uncontrollable impulses to exercise (Lee et al., 2024). Prior research has shown that passion is the ability to drive individuals to sustain their engagement in sports activities, whether out of a love of the sport itself or the need to pursue personal fulfillment, and that this strong inclination not only promotes sustained participation in exercise, but also increases athletic performance and psychological well-being (Li, 2010; Schellenberg et al., 2021). However, existing research has failed to explicitly explore how dualistic passions play a potential mediating mechanism in the relationship between SOP and exercise participation among college students. This study is based on the Theory of Planned Behavior, which suggests that personality traits influence attitudes and behavioral intentions, which ultimately shape behavior (Potwarka, 2015). In this framework, college students who exhibit SOP may set high exercise goals and strive for perfection in their pursuit of personal performance and achievement. This may lead to the development of both harmonious and obsessive passions for exercise, ultimately promoting their exercise participation.

This study presents a new perspective to understand college students’ exercise participation. First, this study investigates the relationship between SOP and exercise participation based on the theory of planned behavior. This not only enriches the application of the theory of planned behavior in different domains, but also provides a new perspective to understand the relationship between personality traits and motor behavior. Second, by examining the role of harmonious and obsessive passions in the relationship between SOP and exercise participation, this study provides a deeper understanding and a new theoretical framework for examining how personality traits influence exercise participation.

## 2 Theoretical framework and hypotheses

### 2.1 Self-oriented perfectionism and exercise participation

Perfectionism is a personal character trait in which individuals set high standards and rigorously evaluate behaviors in the pursuit of perfection (Flett and Hewitt, 2020). Perfectionism is a multifaceted concept that includes SOP. SOP is a personality trait characterized by setting high goals for oneself, being strict with oneself, and criticizing one’s own behavior if one fails to achieve them (Hewitt et al., 2020). This trait of perfectionism encourages individuals to set higher personal goals and increases their motivation and self-discipline to achieve them (Oh, 2019). Goal-setting theory (GST) suggests that specific and challenging goals can significantly increase an individual’s motivation and performance (Locke and Latham, 2002). According to goal-setting theory, college students with SOP tend to set high standards and strive for perfection in exercise participation, and such specific and challenging goals may promote their efforts and improve their exercise participation to reach goals. Prior research has shown that athletes with SOP strive for perfection in sports, and have improved athletic performance (Nordin-Bates et al., 2024). Based on goal-setting theory and prior research, we believe that college students who strive for self-perfection in sports will invest a great deal of time and effort in improving their exercise participation to achieve the high standards and goals they set for themselves. Therefore, we hypothesize the following:

*Hypothesis 1*: SOP is positively related to exercise participation.

### 2.2 Mediating effects of harmonious and obsessive passion

Passion is an individual’s intense fondness and affinity for activities (e.g., sports) that they believe define their self-identity (Vallerand et al., 2003). In other words, when people feel passionate about an activity, it means that they enjoy it immensely and see it as somehow an important part of their identity and self-concept. Vallerand (2020) propose that developing enthusiasm for an activity involves two processes: firstly, assessing the activity, including personal interest, its value, and the emotional experience it brings, and secondly, incorporating the activity into one’s personal identity. Passion is presented as either harmonious passion or obsessive passion through the dualistic development of the internalization process (the extent to which the activity becomes part of the individual’s self-identity) (Vallerand, 2010). Specifically, harmonious passion is an individual’s strong desire and positive emotional engagement to perform a specific activity (Jang et al., 2023). When college students have a harmonious passion for a sport, they are pleased and satisfied by the sport itself, and this pleasure and identification makes them more willing and happy to participate in the exercise participation (Vallerand and Verner-Filion, 2020). Previous research has shown that when individuals participate in exercise out of personal interest, they experience more positive emotions and a sense of achievement (Annenkova et al., 2021), and this positive emotional state not only makes the exercise experience more enjoyable, but also motivates individuals to continue their exercise participation (Vallerand et al., 2003). Pérez García and Guzmán Luján (2019) found that harmonious passion promotes an individual’s intention to exercise.

In contrast, obsessive passion is a process in which an individual internalizes an activity into their self-identity due to internal or external pressures (Vallerand et al., 2023). This internalization may stem from a reliance on a sense of self-worth or social acceptance, or because the excitement from the activity itself is too strong (Vallerand, 2008). In this scenario, college students with an obsessive passion for exercise will prioritize exercise, devote more time to it, and even set aside or neglect other important activities (Bureau et al., 2019). Bonneville-Roussy et al. (2011) argued that individuals with obsessive passions who engage in such activities that are desirable (e.g., health behaviors) motivate individuals to actively participate. Based on the theory of planned behavior, individuals tend to engage in behaviors that they perceive to be generally beneficial (Ajzen, 1991; Ajzen and Albarracín, 2007). In the context of exercise, obsessive passion can serve as a key intrinsic motivator for individuals to engage in exercise when their exercise goals align with their long-term health interests or personal achievement goals, thereby promoting exercise participation.

According to theory of planned behavior, individual personality traits can affect behavioral intentions and behaviors through attitudes and beliefs that influence behavior (Potwarka, 2015). College students with SOP traits have a strong drive to achieve high personal standards and assert their self-worth (Hill et al., 2018), and in athletic situations, this personality trait can facilitate exercise participation through exercise-influenced attitudes (harmonious passion and obsessive passion). Specifically, individuals with SOP traits have extremely high standards for the self, and when they strive to meet these standards, the positive emotions that come with exercise motivate them to be more focused and committed to these activities, promoting a harmonious passion in the individual, which leads to higher levels of exercise participation. On the other hand, when college students with SOP traits closely associate their personal identity, accomplishments, and exercise, this association may lead to college students’ reliance on the success and recognition that comes with exercise participation, which can promote the obsessive passion for exercise, demonstrate high levels of motivation and perseverance (Barr et al., 2015), and promote exercise participation. Based on this, this study proposes the hypothesis:

*Hypothesis 2*: Harmonious passion mediates the relationship between SOP and exercise participation.

*Hypothesis 3*: Obsessive passion mediates the relationship between SOP and exercise participation.

### 2.3 Participants and procedures

This study utilized the professional data collection platform “Credamo” to conduct a questionnaire survey targeting college students, employing a “snowball” sampling method. First, the researchers invited 50 college students around them to participate in the survey and asked these participants to forward the link to the questionnaire to their college friends. This process was continued until data saturation. The questionnaire was distributed via WeChat, China’s main social media platform. The questionnaire was designed to include basic information on SOP, Harmonious passion, Obsessive Passion, exercise participation, and demographics. To preclude data duplication, the system was configured to permit each IP address to access the survey link only once. Furthermore, a 7-point Likert scale attention check question (instructing respondents to select ‘Strongly Agree’) was incorporated into the questionnaire to safeguard data quality.

Drawing on previous studies (Ahmed et al., 2021; Pellerine et al., 2022), the inclusion criteria for this study were as follows: (1) Current enrollment in an undergraduate institution. (2) Ages between 18 and 22 years, aligning with the typical age range of Chinese college students (Naseer and Rafique, 2021).(3) Voluntary consent for participation. (4) Regular exercise participation for a minimum of 2 months (Yuan et al., 2022).(5) Absence of diseases preventing physical activity (Pan et al., 2022). The exclusion criteria were as follows: (1) Exceeding the specified age range. (2) Incomplete or improperly filled questionnaires. (3) Failure to pass the attention check (Shamon and Berning, 2019). (4) Survey completion time less than 2 min. (5) Answers are illogical or patterned(Meterko et al., 2015). (6) Presence of missing data. Through these rigorous screening criteria, 23 questionnaires failing the attention check and 15 questionnaires not meeting the inclusion criteria were excluded. The final valid sample comprised 374 students from 15 provinces, including Beijing, Tianjin, Hebei, and Jiangsu, with 198 females (52.9%) and 176 males (47.1%), and a mean age of 20.39 years (SD = 1.7). The survey included 96 college students (25.7%) in the first year, 97 students (25.9%) in the second year, 110 students (29.4%) in the third year, and 71 students (19%) in the fourth year.

This study implemented pilot testing, data cleaning, and reliability and validity tests to ensure data completeness and reliability. Specifically, two rounds of pilot tests were conducted, and based on the feedback received, the questionnaire was subsequently adjusted and refined (Aftab et al., 2023). Secondly, strict data cleaning was conducted according to the inclusion and exclusion criteria to ensure the quality of the analyzed data. Finally, the scales employed in the study were tested for reliability (Cronbach’s alpha) and validity (confirmatory factor analysis: CFA) to ascertain robustness.

All participants voluntarily took part in the survey and were informed that it was anonymous and confidential. Electronic consent was obtained from all participants for the study. The study procedures were conducted in accordance with the ethical standards of the Chinese Psychological Association and the 1964 Declaration of Helsinki, as well as its subsequent amendments or similar ethical standards. This study (2024011204) was approved by the Ethics Committee of Tianjin Normal University.

### 2.4 Measures

The scale used in this study was originally in English, and based on Brislin (1986) suggestion, a translation and back-translation method was used to convert it into Mandarin and then translate it back into English to ensure clarity of the measurement tool. The questionnaire was improved in the first and second rounds of pilot testing with 20 and 25 participants, respectively. The main purpose of the test is to identify items that are not clearly expressed in Mandarin. Comments from participants in the first round indicated that some items were ambiguous, and we amended these items to improve their accuracy. A second round of testing confirmed the complete clarity of the questionnaire.

#### 2.4.1 Self-oriented perfectionism

The SOP scale of the Multidimensional Perfectionism Scale (MPS) developed by Hewitt and Flett (1991) was used in this study. The scale is a one-dimensional structure with 15 items (e.g., “My goal is to be perfect in everything”), each of which has a 7-point scale ranging from 1 (not at all) to 7 (perfectly), with the higher the total score, the higher the individual’s degree of SOP (Cronbach’s α = 0.91).

#### 2.4.2 Passion

The present study used the Passion Scale developed by Vallerand et al. (2003) to measure the level of sports passion among college students. The scale consists of 2 subscales, Harmonious Passion and Compulsive Passion, with a total of 14 items (e.g., “The new things I get from sport give me more value”), and the questionnaire is scored on a 7-point Likert scale. In the reliability test, the Cronbach’s α coefficient was 0.93 for the total scale and 0.96 and 0.91 for the subscales.

#### 2.4.3 Exercise participation

The exercise participation scale used by Ahn et al. (2016) was adopted for this study. The scale consists of three questions. Specific entries included the question, “How much time do you spend on physical activity each day, other than school physical education classes?” with response scales of 1 (no participation or less than 30 min), 2 (30 min - 1 h), 3 (1 h - 2 h), and 4 (more than 2 h). “How often do you participate in physical activity in a week?” with a response scales of 1 (no participation or 1 time), 2 (2–3 times a week), 3 (4–5 times a week), and 4 (6–7 times a week). “If you have participated in physical activity, for how long?” with response scales of 1 (no participation or less than 3 months), 2 (3 months - 6 months), 3 (6 months - 1 year), and 4 (more than 1 year). Each item was rated on a 4-point Likert scale, with higher scores indicating greater levels of exercise participation (Cronbach’s α = 0.75).

#### 2.4.4 Data analysis strategy

After collecting the questionnaire data, the study was processed using the following methods: Firstly, CFA was executed using Mplus 8.3 software. Secondly, the reliability of the scale was assessed by Cronbach’s α coefficient and tested for discriminant validity using Fornell and Larcker (1981) criteria. In addition, a Harman’s one-factor test was conducted to detect common method bias (Podsakoff et al., 2012). Finally, the significance of the indirect effect of harmonious passion versus obsessive passion was tested using a bootstrap sample of 5,000 and 95% bias-corrected confidence intervals to ensure that the interval of significant indirect effects did not contain a zero (Preacher and Hayes, 2008).

## 3 Results

### 3.1 Reliability and validity

Prior to formulating the hypotheses, this study conducted a CFA on the following variables: SOP, harmonious passion, obsessive passion, and exercise participation as a means of assessing the fit of the measurement model. The results of the analyses showed a good fit for the four factors as follows: *χ*^2^/df = 2.37, CFI = 0.93, TLI = 0.92, RMSEA = 0.06, and SRMR = 0.04.

The lowest value of Cronbach’s alpha coefficient in the study was 0.75, indicating good reliability of the scale. In addition, convergent validity was satisfactory, with the combined reliability (CR) of each construct ranging from 0.75 to 0.96 (CR > 0.70). The mean variance extracted for each construct ranged from 0.51 to 0.76 (AVE > 0.50), which all exceeded the suggested threshold, showing good discriminant validity as the square root of the AVE was greater than the correlation of all factors. In addition, this study used Harman’s one-factor test to test the presence of common method bias in the collected data (Podsakoff et al., 2012), which showed that the variance explained by the rotated first factor was 41.22%, which is less than 50% indicating that the data in this study did not have a significant common method bias.

### 3.2 Descriptive statistics and correlation analysis

Descriptive statistical analysis of demographic information and research variables were conducted in this study to capture the basic characteristics of the data. In addition, correlation analysis was conducted to gain a deeper understanding of the potential links between the variables. The results of the analysis are shown in Table 1. The results showed significant positive correlations between SOP and harmony passion (*r* = 0.45, *p* < 0.01), obsessive passion (*r* = 0.46, *p* < 0.01), and exercise participation (*r* = 0.47, *p* < 0.01). Furthermore, there was a significant positive correlation between passion for harmony and exercise participation (*r* = 0.48, *p* < 0.01). Similarly, there was a significant positive correlation between obsessive passion and exercise participation (*r* = 0.53, *p* < 0.01).

**Table 1:** Descriptive statistics, correlations, and reliabilities.

Variables	*M*	*SD*	1	2	3	4	5	6	7
1. Gender	1.53	0.50	1						
2. Age	20.39	1.17	−0.01	1					
3. Class	2.42	1.07	−0.03	0.49**	1				
4. Self-oriented perfectionism	4.69	0.98	0.04	0.02	0.04	1			
5. Harmonious passion	3.80	1.37	0.06	0.05	−0.05	0.45*	1		
6. Obsessive passion	3.68	1.18	−0.01	0.10	0.02	0.46**	0.44**	1	
7. Exercise participation	2.29	0.74	−0.07	0.03	0.03	0.47**	0.48**	0.53**	1

*N* = 374. Gender = “male” (1), “Female” (2); Class = “first year” (1), “second year” (2), “third year” (3), “fourth year” (4).*indicates statistical significance at the *p* < 0.05 level. **indicates statistical significance at the *p* < 0.01 level.

### 3.3 Structural equation modeling test

**Figure 1:**
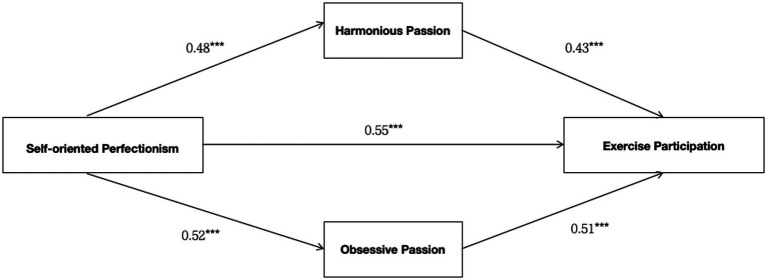
Parallel intermediation model.

After confirming the existence of correlation between all the main variables, this study used Mplus 8.3 to build structural equation modeling to further analyze the relationship between the variables, and the results are shown in Table 2 and Figure 1. To test Hypothesis 1, the analyses revealed a significant positive effect of SOP on exercise participation (β = 0.55, *p* < 0.001; see Model 3). The results support hypothesis 1. Model 1 and model 4 results showed a significant positive effect of SOP on exercise participation through harmonious passion (β = 0.16, *p* < 0.001, 95% C.I = [0.90, 0.25]), suggesting a complementary mediating effect (Zhao et al., 2010). The results support hypothesis 2. The results of Model 2 and Model 5 showed that SOP had a significant positive effect on exercise participation through obsessive passion (β = 0.20, *p* < 0.001, 95% C.I = [0.14, 0.29]), suggesting a complementary mediating effect (Zhao et al., 2010). The findings support Hypothesis 3. In addition, this study employed the Sobel test to assess the mediating effect’s significance. The results of the analysis showed a significant mediation effect of harmonious passion between SOP and exercise participation (*z* = 4.68, *p* < 0.001). The mediation effect of obsessive passion between recognition of SOP and exercise participation was significant (*z* = 6.90, *p* < 0.001).

**Table 2:** Mediating role of harmonious and obsessive passions.

Dependent variables	Harmonious passion		Obsessive passion		Exercise participation					
	Model 1		Model 2		Model 3		Model 4		Model 5	
Measure	β (*SE*)	*p*	β (*SE*)	*p*	β (*SE*)	*p*	β (*SE*)	*p*	β (*SE*)	*p*
1.Gender	0.03 (0.05)	0.570	−0.05 (05)	0.374	−0.10 (0.05)	0.054	−0.11 (05)	0.022	−0.08 (05)	0.105
2. Age	0.12 (0.05)	0.032	0.16 (06)	0.006	0.09 (0.06)	0.162	0.04 (0.06)	536	0.01 (0.06)	0.890
3. Class	−0.13 (0.05)	0.015	−0.08 (0.06)	0.177	−0.04 (0.06)	0.546	0.02 (0.06)	0.763	0.00 (0.06)	0.968
4. Self-oriented perfectionism	0.48 (0.05)	0.000	0.52 (0.06)	0.000	0.55 (0.05)	0.000	0.35 (0.08)	0.000	0.29 (05)	0.000
5. Harmonious passion							0.43 (0.08)	0.000		
6. Obsessive passion									0.51 (0.05)	0.000
*R^2^*	0.24		0.29		0.32		0.46	0.50		
*ΔR* ^2^	0.23		0.28		0.31		0.45	0.49		
*F*	16.51^***^		21.36^***^		58.04^***^		105.06^***^	123.33^*^		

*N* = 374. β is standardized regression coefficients, SE is the standard error. Bootstrap sample size = 5,000. gender = “male” (1), “female” (2).*indicates statistical significance at the *p* < 0.05 level. ***indicates statistical significance at the *p* < 0.001 level.

## 4 Discussion

Based on the theory of planned behavior, we conducted hypothesis testing and found supportive evidence that college students’ SOP directly influences exercise participation. It also indirectly increases levels of exercise participation through harmonious passion or obsessive passion. Below, we discuss the theoretical and practical implications of these results, the limitations of the study, and directions for future research.

### 4.1 Theoretical implications

This study provides a theoretical contribution to understanding college students’ exercise participation behaviors. Firstly, this study extends existing research on factors influencing exercise participation. Existing research has focused on the effects of an individual’s gender, education, social and psychological factors on sport participation (Li et al., 2023), while less research has been conducted on the role of individual personality traits, particularly SOP, in exercise participation. The results of the present study demonstrated that SOP tendency is one of the important predictors of college students’ sports participation, filling the gap of the effect of SOP tendency on sports participation that has not been investigated in the prior studies.

Second, the results of this study suggest that harmonious passion and obsessive passion mediate the relationship between SOP and exercise participation. This finding provides empirical support for the dichotomous model of passion in sport psychology, revealing a complex mechanism for the role of passion in the relationship between SOP and exercise participation. Consistent with the theory of planned behavior (Ajzen, 1991), the results of this study suggest that both harmonious passion and obsessive passion can be used as attitudes to elucidate the link between SOP and participation in exercise. Harmonious Passion provides value to participation in the activity itself and allows college students to participate more autonomously (Yukhymenko-Lescroart, 2021). The results of this study support previous findings. Consistent with Lee et al. (2023), harmonious passion plays a significant mediating role between self-determined motivation and athletes’ commitment to exercise, and athletes’ self-determined motivation is enhanced by harmonious passion, which influences their continued exercise participation and commitment to the sport and improves performance and satisfaction (Gu et al., 2022). In addition, Yukhymenko-Lescroart (2021) showed that harmonic passion acts as a mediator to enhance individual athletic performance and psychological well-being, positively regulating athletes’ psychological state and behavioral performance. Therefore, college students with SOP can also enjoy exercise by developing an intrinsic interest in it and positive emotions, which can increase their exercise participation.

Unlike harmonious passion, obsessive passion stems from internal and external pressures and even insecurity about self-worth (Vallerand, 2008). Most prior research has focused on the possibility that obsessive passion may cause negative outcomes for individuals such as negative emotions or fatigue (Curran et al., 2011). For example, Lopes and Vallerand (2020) found that the higher an athlete’s obsessive passion, the higher the burnout. However, our findings suggest that obsessive passion plays a motivational role in exercise, increases college students’ exercise participation, and positively mediates the relationship between SOP and exercise participation. This result suggests that that college students with SOP trait may be compelled to internalize exercise participation due to the pressure to be perfect, increasing the individual’s obsessive passion for exercise and increasing the level of exercise participation. The results of this study are consistent with the study by Dalla Rosa and Vianello (2020), demonstrating the positive mediating effect of obsessive passion. By revealing this complex mechanism, this study enriches the study of obsessive passion and contributes to a comprehensive understanding of obsessive passion.

### 4.2 Practical implications

Our study provides some implications for physical educators to promote exercise participation among college students. Firstly, we found that college students with a higher SOP have higher levels of exercise participation due to their pursuit of high standards and perfection. This suggests that not only athletes (Waleriańczyk and Stolarski, 2021), but also college students can benefit from higher levels of SOP in terms of sports participation. Based on this finding, physical educators should encourage and guide college students with high SOP tendencies to exercise their pursuit of high standards and excellence and transform this drive into positive motivation to participate in the exercise. For example, physical educators can incorporate interactive goal-setting sessions into their physical education programs to encourage students to set achievable goals for themselves in physical activity.

Second, our study identified a critical positive mediating effect of harmonious and obsessive-compulsive passions between SOP and exercise participation. This suggests that by fostering a harmonious passion, physical educators can help students transform their intrinsic drive for perfection into a driving force for active participation in physical activity, facilitating participation in exercise in a healthier, more balanced way. Specifically, we recommend designing interventions in universities that encourage students to develop a passion for harmony and apply it to physical activity. For example, physical education programs and activities could be designed to be more flexible and varied, giving students the opportunity to explore different types of sports and to find activities that they truly enjoy. University students could also be encouraged to play sports in a group setting to enhance harmonious passion through social interaction and group identity.

Finally, for current college students, SOP has a facilitating effect on exercise participation through obsessive passion. This implies that perfection-seeking students may participate in exercise more frequently due to obsessive passion, and that they see exercise as a means of realizing their personal values, and so show greater commitment and persistence in exercise participation. Therefore, it is crucial for university students to have a deep understanding of their behavioral motivations and emotional states. Teachers need to set more realistic and balanced goals for their college students, with athletic goals that are challenging but achievable, and to teach college students effective time management skills to ensure that they have enough time for study, leisure, socializing and rest.

### 4.3 Limitations and future recommendations

This study has several limitations. First, this study was only a cross-sectional study of Chinese college students, which has some limitations in terms of the generalizability of the study and the causality of the variables. Therefore, future studies should adopt a longitudinal design, experimental intervention, or control group approach to more accurately and effectively explore the causal relationship between SOP and exercise participation.

Second, considering that this study was limited to a specific population of Chinese university students, the generalizability of the findings may be limited. Future studies should also expand the scope of sample selection to include college students of different cultural backgrounds or other groups to enhance the generalizability and representativeness of the findings.

Third, this study only examined Chinese college students’ SOP and failed to examine whether the perfectionist tendencies of other-oriented perfectionism and socially prescribed perfectionism had an impact on college students’ exercise participation. Individuals may react differently to actions due to different perfectionist tendencies. For example, socially prescribed perfectionism assumes that significant others hold high expectations of themselves and that others pressure themselves for perfection (Verner-Filion and Vallerand, 2016). This socially prescribed perfectionism may be a pressure or a motivator for college students’ exercise participation, and future research could explore how this external motivation may affect exercise participation.

Fourth, Although the results of Harman’s one-factor test showed that CMB was not a serious problem in this study, this value (41.22%) was relatively high. This suggests that common method bias may still be a potential problem. Future research should consider employing multiple data collection methods, such as cross-validating findings through longitudinal designs or utilizing diverse data sources, to further mitigate the risk of common method biases and to more robustly substantiate findings.

## Data availability statement

The raw data supporting the conclusions of this article will be made available by the authors, without undue reservation.

## Ethics statement

The studies involving humans were approved by Ethics Committee of Tianjin Normal University. The studies were conducted in accordance with the local legislation and institutional requirements. Written informed consent to participate in this study was not required from the participants in accordance with the national legislation and the institutional requirements.

## Author contributions

WZ: Data curation, Software, Writing – original draft, Writing – review & editing. YZ: Conceptualization, Data curation, Methodology, Writing – review & editing. FJ: Data curation, Methodology, Writing – review & editing. HS: Investigation, Software, Writing – review & editing.
